# A Novel Obstacle Traversal Method for Multiple Robotic Fish Based on Cross-Modal Variational Autoencoders and Imitation Learning

**DOI:** 10.3390/biomimetics9040221

**Published:** 2024-04-08

**Authors:** Ruilong Wang, Ming Wang, Qianchuan Zhao, Yanling Gong, Lingchen Zuo, Xuehan Zheng, He Gao

**Affiliations:** 1 School of Information and Electrical Engineering, Shandong Jianzhu University, Jinan 250101, China; 2 Department of Automation, Tsinghua University, Beijing 100084, China; 3 Shandong Zhengchen Technology Co., Ltd., Jinan 250101, China

**Keywords:** multiple robotic fish, visual navigation, CM-VAE, imitation learning

## Abstract

Precision control of multiple robotic fish visual navigation in complex underwater environments has long been a challenging issue in the field of underwater robotics. To address this problem, this paper proposes a multi-robot fish obstacle traversal technique based on the combination of cross-modal variational autoencoder (CM-VAE) and imitation learning. Firstly, the overall framework of the robotic fish control system is introduced, where the first-person view of the robotic fish is encoded into a low-dimensional latent space using CM-VAE, and then different latent features in the space are mapped to the velocity commands of the robotic fish through imitation learning. Finally, to validate the effectiveness of the proposed method, experiments are conducted on linear, S-shaped, and circular gate frame trajectories with both single and multiple robotic fish. Analysis reveals that the visual navigation method proposed in this paper can stably traverse various types of gate frame trajectories. Compared to end-to-end learning and purely unsupervised image reconstruction, the proposed control strategy demonstrates superior performance, offering a new solution for the intelligent navigation of robotic fish in complex environments.

## 1. Introduction

Object detection, tracking, and traversal using visual-based approaches have been extensively studied in the field of computer vision. These methods have been applied in diverse domains, including robot navigation [1], traffic monitoring [2], and crowd detection [3]. The utilization of visual approaches across many platforms is a significant advancement in harnessing the potential of growing visual detection technologies. Current robotic platforms include underwater robots, surface vessels, ground-based robots, and unmanned aerial aircraft, along with other intelligent technologies. The research and utilization of several robotic platforms have greatly improved the capacity for environmental investigation. There is currently a significant increase in the interest around the examination of underwater robotic platforms, which have a wide range of practical uses. The combination of visual technology with underwater robotic platforms has the potential to accelerate technological progress in both fields, consequently increasing their societal usefulness [4].

Robotic fish, a sort of underwater robot, have the ability to perform underwater reconnaissance and mobile monitoring of environmental targets [5]. Underwater robot platforms are commonly used for the navigation of target obstacles by several robotic fish. GPS navigation systems are frequently necessary to furnish navigation and positioning data for robotic fish in such scenarios. Nevertheless, the arduous communication circumstances underwater provide obstacles for robotic fish in establishing effective communication with the surrounding environment, resulting in a decline in the precision of GPS navigation systems [6]. Thus, this work employs a visual navigation strategy to navigate through target impediments.

Regarding the matter of visual navigation, extensive research has been conducted both domestically and internationally, resulting in notable advancements. Popular methods for visual navigation include mapping-based approaches [7], deep reinforcement learning [8], and imitation learning [9]. Within the field of map-based visual navigation research, techniques can be classified into two sub-directions based on the precision and structure of their map construction: metric map-based visual navigation and topological map-based visual navigation [10]. VSLAM-generated maps belong to the metric map category. VSLAM, in theory, is defined as follows: A mobile robot, equipped with visual sensors, constructs a model of the surrounding environment and concurrently estimates its own motion while in motion, without any prior knowledge of the environment [11]. In their study, Wang et al. [12] introduced a technique that utilizes a semantic topological map and ORB-SLAM2 for relocalization and loop closure detection. This strategy significantly enhances the precision of relocalization and loop closure detection in dynamic situations. Although visual SLAM approaches have the capability to produce precise metric maps, the specific demands for localization and mapping include substantial time and labor expenses. The intricacy of these requirements impedes the ability to effectively strategize and navigate in extensive settings.

Visual teach and repeat (VT&R) [13] is a navigation strategy that relies on topological maps. During the teaching phase, also known as mapping, a path is created by gathering a collection of images with the assistance of a human. During the repeating phase, the motion commands are calculated exclusively using topological information or in combination with accurate metric information [14]. The use of deep learning for visual teach and repeat navigation has emerged as an important area of research [15]. Roucek et al. [16] introduced a technique that enables neural networks to be trained autonomously in VT&R tasks, which involve a mobile robot repeating a path it has been taught before. Although neural networks used in teach and repeat navigation can demonstrate resistance to specific alterations in input images, there remains a requirement to improve the network’s ability to manage more extensive fluctuations in the environment.

The research on visual navigation methods based on deep reinforcement learning tackles several challenges, including the scarcity of rewards [17], the intricacy of time [18], and the extensive dimensionality of motion space [19]. Additionally, it addresses the difficulties arising from the large number of training samples and the complex representation of input data [20]. In order to address these difficulties, Ashlesha Akella et al. [21] proposed a temporal framework that integrates dynamic systems and deep neural networks. This paradigm facilitates the understanding of the passage of time (“action time”) and actions, enabling the agent to assess the timing of its actions depending on the input velocity, hence improving the navigation performance. The LS-modulated compound learning robot control (LS-CLRC) approach was proposed by Guo et al. [22]. The LS-CLRC technique ensures that all parameter estimates converge at a similar rate, resulting in a balanced convergence rate for all components of the parameter vector and enhancing the usage of data. The research findings exhibit substantial progress in comparison to prior investigations.

Wu et al. [23] introduced a new MARDDPG algorithm specifically designed to tackle the problem of traffic congestion at many crossings in transportation. The centralized learning within each evaluation network allows each agent to accurately assess the policy execution of other agents while making decisions. The approach utilizes long short-term memory (LSTM) to capture concealed state information and shares parameters in the actor network to accelerate the training process and decrease memory use. Carrillo Mendoza et al. [24] created the robot architecture AutoMiny, which is operated proportionately. Data for training were gathered and the network was evaluated using the NVIDIATM arm architecture graphics processor unit. The researchers developed a Siamese deep neural network structure called the visual autonomous localization network (VALNet) to perform visual localization. This network is specifically designed to estimate the ego-motion of a given sequence of monocular frames.

Bozek et al. [25] proposed an algorithm for training artificial neural networks for path planning. The trajectory ensures optimal movement from the current position of the mobile robot to the specified location, while considering its direction. Segota et al. [26] trained a feedforward type multilayer perceptron (MLP), which can be used to compute the inverse kinematics of robot manipulators. Luís Garrote et al. [27] suggested a localization technique that combines particle filtering, sometimes referred to as Monte Carlo localization, with map updates using reinforcement learning. This method combines measurements that are relative to each other with data obtained from absolute indoor positioning sensors. An inherent characteristic of this localization method is its capacity to modify the map if notable alterations pertaining to the existing localization map are identified. The adaptive Kalman filtering navigation algorithm, abbreviated as RL-AKF, was introduced by Gao et al. [28]. The RL-AKF approach employs deep deterministic policy gradients to estimate the process noise covariance matrix. The reward is defined as the negative value of the current localization error. The method is specifically designed to be used in a navigation system that combines multiple elements, and it may be applied to action spaces that are continuous in nature.

Spurr et al. [29] introduced a technique for acquiring a statistical hand model by creating a deep neural network that captures the hidden space representation through cross-modal training. The goal function is obtained from the variational lower limit of the variational autoencoders (VAE) framework. The optimization process of the target function involves the simultaneous incorporation of the cross-modal KL divergence and posterior reconstruction objectives. This naturally employs a training technique that results in a consistent configuration of latent space across several modalities, including RGB pictures, 2D keypoint detection, and 3D hand representations. These approaches collectively show that deep reinforcement learning methods greatly improve the navigation of mobile robots, allowing them to reach specified places more quickly and precisely.

Wu et al. [30] introduced a goal-driven navigation system for map-free visual navigation in indoor environments, as part of their research on imitation learning in this field. This method utilizes different perspectives of the robot and the target image as inputs during each time interval to produce a sequence of actions that guide the robot towards the objective, hence avoiding the necessity of relying on odometry or GPS during runtime. Due to the lack of safety assurance in learning-based map-free navigation approaches, imitation learning can be utilized to train map-free navigation policies using 2-D LiDAR data in a safe manner. This solution employs an innovative IL training method that relies on dataset aggregation, offering supplementary safety improvements [31]. Behavior cloning, a form of imitation learning, has proven useful in learning basic visual navigation strategies by the imitation of extensive datasets generated from expert driving actions [32]. Additionally, visual navigation imitation learning techniques encompass direct policy learning [33], inverse reinforcement learning [34], and generative adversarial imitation learning (GAIL) [35].

In recent years, underwater robots equipped with advanced intelligent navigation algorithms have demonstrated significant potential for autonomous operations underwater. Zhang et al. [36] proposed a deep framework called NavNet, which treats AUV navigation as a deep sequential learning problem. NavNet exhibits an outstanding performance in both navigation accuracy and fault tolerance, achieving the precise navigation and positioning of AUVs. Ruscio et al. [37] introduced a navigation strategy for underwater surveillance scenarios, which integrates a single bottom-view camera with altitude information for linear velocity estimation. This allows the effective use of payloads already required for monitoring activities, also for navigation purposes, thereby reducing the number of sensors on the AUV. Song et al. [38] presented an acoustic-visual-inertial navigation system (Acoustic-VINS) for underwater robot localization. Specifically, they addressed the issue of global position ambiguity in underwater visual-inertial navigation systems by tightly coupling a long baseline (LBL) system with an optimization-based visual-inertial SLAM. Yan et al. [39] proposed a novel autonomous navigation framework integrating visual stabilization control, based on a navigation network generated from stereo vision. While this approach addresses limitations of underwater visual conditions and restricted robot motion in visual navigation methods, it does not fully tackle the precise control of robot visual navigation in complex underwater environments. This paper presents the precise control of multi-robotic fish crossing obstacle technology based on visual navigation based on deep learning and imitation learning, by introducing a learning framework that needs to learn the cross-modal representation of state coding. The initial data modality consists of unannotated, unprocessed first-person view (FPV) images, whereas the second data modality encompasses state information linked to the gate frame. Annotations are provided during the swimming phase of the robotic fish to indicate the relative attitude of the following gate frame in the FPV. The utilization of a cross-modal variational autoencoder (CM-VAE) architecture allows for the acquisition of a low-dimensional latent representation. This paradigm employs encoder–decoder pairs for each data modality, constraining all inputs and outputs that enter and exit a unified latent space. Therefore, it is possible to include both labeled and unlabeled data in the training procedure of latent variables. Afterwards, imitation learning is utilized to train a control policy that maps the latent variables to velocity commands for the robotic fish.

The innovation of this study lies in the following:

(i) Incorporating the CM-VAE architecture into the navigation system of robotic fish enables the effective processing of diverse modal data. This allows information from different modalities to be seamlessly integrated and expressed within a unified framework, thereby enhancing the overall perception and comprehension capabilities of the robotic fish towards its environment.

(ii) The integration of CM-VAE’s feature extraction and imitation learning navigation strategies provides real-time optimization for the motion planning of multiple robotic fish. This facilitates a gradual learning process for the robotic fish to handle intricate navigation tasks, while concurrently accounting for the influence of multimodal perception data, thereby enhancing the stability and generalization capabilities of the learning process.

(iii) The application of CM-VAE and imitation learning on the Unreal Engine simulation platform has realized a realistic virtual simulation environment. This enables multiple robotic fish to learn in the simulation, facilitating improved generalization to the real world and providing a reliable training and validation environment for practical applications.

The remaining of the paper are organized as follows: Section 2 provides a comprehensive description of the fundamental structure of the control system for the robotic fish. The first-person view (FPV) of the robotic fish is transformed into a compressed representation in a low-dimensional latent space using CM-VAE. The control strategy then translates into velocity directives for the robotic fish. The CM-VAE design incorporates an encoder–decoder pair for each data modality, and the control policy in imitation learning leverages behavior cloning. Section 3 presents the introduction of the experimental platform and environment. The efficacy of the proposed visual navigation system is confirmed by trials in which robotic fish successfully navigate across various gate paths. Section 4 presents the empirical findings of robotic fish navigating through different gates. It also highlights the constraints of the visual navigation technique and proposes potential avenues for future research. Section 5 of the study provides a concise overview of the primary content and experimental findings. It also evaluates the advantages and constraints of visual navigation techniques and finishes by detailing the forthcoming research directions.

## 2. Cross-Modal Control Strategy

This study focuses on the task of enabling a robotic fish to navigate independently across gate frames in the underwater environment simulation platform of the Unreal Engine (UE). The research is focused on two primary areas: (i) constructing a cross-modal variational autoencoder architecture, and (ii) establishing a control method using imitation learning to manage the latent space.

### 2.1. Control System Framework

**Figure 1:**
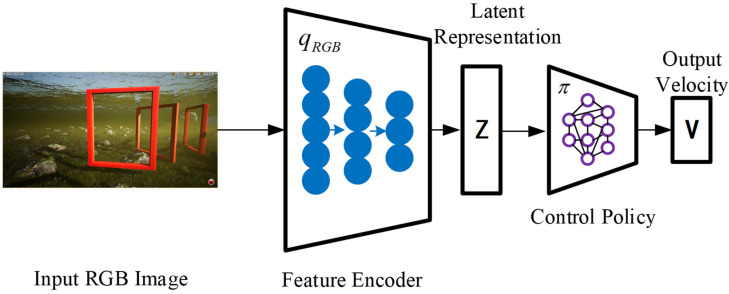
Control system architecture. Input images are encoded into low-dimensional latent representations, and the control strategy obtains desired output velocity commands through this representation.

The front camera component of the UE receives images, which serve as the first-person view (FPV) for the robotic fish. The low-dimensional latent representation encodes the relative attitude to the next visible gate frame, as well as the background information. Afterwards, the hidden representation is inputted into the control network, which generates velocity commands. The motion controller of the robotic fish translates these commands into actuator commands, as shown in Figure 1, which depicts the system framework.

In this system workflow, it is necessary to collect image data of robotic fish during the obstacle traversal task of passing through gate frames. Utilizing the collected data, a cross-modal VAE model is trained. This model effectively captures the correlations between multimodal data and encodes them into a representation in a latent space. Based on imitation learning, the behavioral strategy of the robotic fish during the obstacle traversal task is trained. Expert data are employed for training an imitation learning model, enabling it to predict the correct actions from given observation states. The trained imitation learning model can be used to generate behavioral strategies for robotic fish during the obstacle traversal task. When facing new environments, the predicted actions from the model can be utilized to execute tasks.

### 2.2. Robotic Fish Kinematic Model

**Figure 2:**
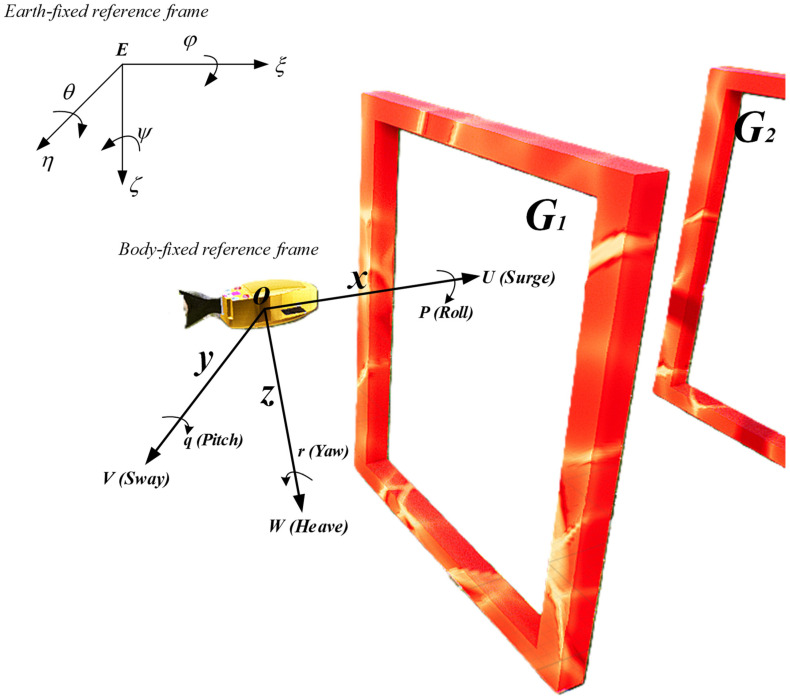
Schematic diagram of robotic fish motion.

When establishing the motion equation model for robotic fish, the inertial coordinate system and the fish body coordinate system are typically employed to analyze the motion of the robotic fish. As illustrated in Figure 2, E−ξηζ represents the inertial coordinate system, while O−xyz represents the body coordinate system. With the transformation relationship between the two coordinate systems, position variables can be calculated from known velocity variables, which constitutes the primary focus of research in robotic fish kinematics.

In the process of studying the spatial motion equations of robotic fish, it is common practice to place the origin O of the fish body coordinate system at the centroid of the robotic fish. The spatial position of the robotic fish is determined by three coordinate components (ξ,η,ζ) in the inertial coordinate system, along with angular components (φ,θ,ψ). The components (u,v,w) represent the velocity components of the fish body coordinate system, while (p,q,r) represent the angular velocity components of the fish body coordinate system.

When the origin of the E−ξηζ coordinate system coincides with the O−xyz coordinate system, according to Euler’s theorem, rotations are performed in the order of ψ→φ→θ. After three consecutive rotation transformations of the coordinate vectors in the O−xyz coordinate system, they can coincide with the vectors in the E−ξηζ coordinate system. Subsequently, the velocity vector of the robotic fish at point O−xyz in the fish body coordinate system is denoted as ${\left(u\;v\;w\right)}^{T}$, which is transformed into coordinates ${\left(\dot{\xi }\;\dot{\eta }\;\dot{\zeta }\right)}^{T}$ in the inertial coordinate system E−ξηζ. According to the principles of coordinate transformation, the following velocity transformation relationship can be obtained as shown in Equation (1):

(1) $$\left[\begin{array}{c} \dot{\xi }\\ \dot{\eta }\\ \dot{\zeta } \end{array}\right]=S\left[\begin{array}{c} u\\ v\\ w \end{array}\right]$$

In the above equation, the transformation matrix S is defined as shown in Equation (2):

(2) $$S^{-1}=S^{T}=\left[\begin{array}{ccc} \cos\psi \cos\theta & \sin\psi \cos\theta & -\sin\theta \\ \cos\psi \sin\theta \sin\varphi -\sin\psi \cos\varphi & \sin\psi \sin\theta \sin\varphi +\cos\psi \cos\varphi & \cos\theta \sin\varphi \\ \cos\psi \sin\theta \cos\varphi +\sin\psi \sin\varphi & \sin\psi \sin\theta \cos\varphi -\cos\psi \sin\varphi & \cos\theta \cos\varphi \end{array}\right]$$

Similarly, if the angular velocity vectors of the robotic fish in the inertial coordinate system are denoted as $\dot{\Lambda }={\left(\dot{\varphi }\;\dot{\theta }\;\dot{\psi }\right)}^{T}$, and in the body coordinate system are denoted as $\Omega ={\left(p\;q\;r\right)}^{T}$, then the transformation relationship can be derived as shown in Equation (3):

(3) $$\begin{array}{c} \dot{\Lambda }=C\Omega \\ C=\left[\begin{array}{ccc} 1 & \sin\varphi \tan\theta & \cos\varphi \tan\theta \\ 0 & \cos\varphi & -\sin\varphi \\ 0 & \sin\varphi /\cos\theta & \cos\varphi /\cos\theta \end{array}\right] \end{array}$$

Combining the above, let the position vector of the robotic fish be denoted as $\eta ={\left[\xi ,\eta ,\zeta ,\varphi ,\theta ,\psi \right]}^{T}$n and the velocity vector be denoted as $v={\left[u,v,w,p,q,r\right]}^{T}$. Therefore, the vector form of the robotic fish kinematic model can be obtained as shown in Equation (4):

(4) $$\begin{array}{c} \dot{\eta }=J(\eta )v\\ J(\eta )=\left[\begin{array}{cc} S & 0_{3\times 3}\\ 0_{3\times 3} & C \end{array}\right] \end{array}$$

Expanding the kinematic vectors, the kinematic model of the robotic fish can be obtained as shown in Equation (5):

(5) $$\left\{\begin{array}{l} \dot{\xi }=u\cos\psi \cos\theta +v(\cos\psi \sin\theta \sin\varphi -\sin\psi \cos\varphi )+w(\cos\psi \sin\theta \cos\varphi +\sin\psi \sin\varphi )\\ \dot{\eta }=u\sin\psi \cos\theta +v(\sin\psi \sin\theta \sin\varphi +\cos\psi \cos\varphi )+w(\sin\psi \sin\theta \cos\varphi -\cos\psi \sin\varphi )\\ \dot{\zeta }=-u\sin\theta +v\cos\theta \sin\varphi +w\cos\theta \cos\varphi \\ \dot{\varphi }=p+q\sin\varphi \tan\theta +r\cos\varphi \tan\theta \\ \dot{\theta }=q\cos\varphi -r\sin\varphi \\ \dot{\psi }=q\sin\varphi /\cos\theta +r\cos\varphi /\cos\theta \end{array}\right.$$

### 2.3. Cross-Modal VAE Architecture

Unsupervised learning is a machine-learning technique that operates without the need for labeled datasets. This method is frequently utilized for the purposes of data clustering, dimensionality reduction, and feature extraction. The VAE, or variational autoencoder, is a deep-learning model designed to enhance the process of learning data representations in unsupervised learning settings. By using the benefits of both generative adversarial networks (GAN) and autoencoders, a variational autoencoder (VAE) is capable of producing top-notch data samples and representing data in a space with fewer dimensions.

An efficient method for reducing dimensionality should possess the qualities of smoothness, continuity, and consistency [40]. In order to accomplish this objective, this article utilizes the CM-VAE framework, which employs an encoder–decoder pair for each data modality, while restricting all inputs and outputs to a latent space.

The overall architecture of CM-VAE is as follows:

1. Encoder: For each modality, there is a corresponding encoder network responsible for encoding the input data into mean and variance parameters in the latent space. The encoder network can be a multi-layer neural network or a neural network structure specific to the modality;
2. Latent space sampling: Based on the mean and variance parameters output by the encoder, a latent vector is sampled from the latent space. This latent vector represents the representation of the input data in the latent space;
3. Decoder: Similarly, for each modality, there is a corresponding decoder network responsible for decoding the latent vector into the reconstruction of the original data. The decoder network can also be a multi-layer neural network corresponding to the encoder.

**Figure 3:**
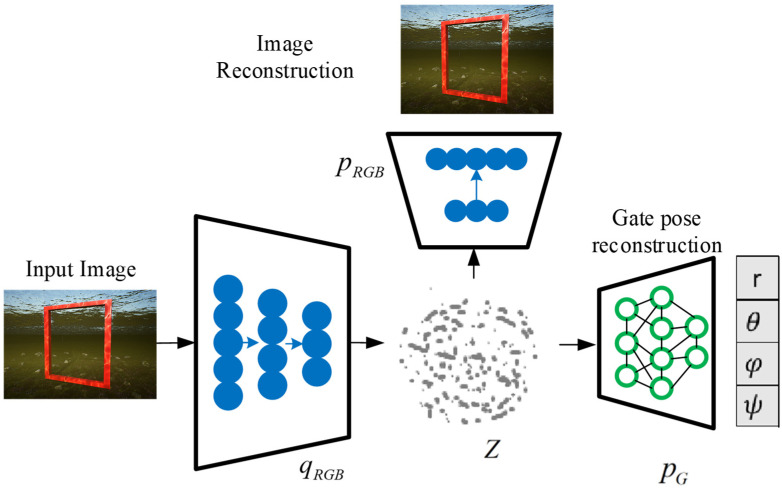
Cross-modal architecture. Each data sample is encoded into a latent space, which can be decoded back into an image or transformed into another data modality, such as the pose of the robotic fish relative to the gate frame.

In the context of robotic fish visual navigation, the data modalities are defined as RGB images and the relative pose of the next gate frame to the robotic fish frame. The input data from the first-person perspective of the robotic fish are processed by the image encoder $q_{RGB}$, forming a latent space with a normal distribution $N(\mu_{t},\sigma_{t}^{2})$, from which $z_{t}$ is sampled. The data modalities can be reconstructed from the latent space using the image decoder $p_{RGB}$ and the gate pose decoder $p_{G}$, as shown in Figure 3.

In the standard definition of VAE, the objective is to learn the probability distribution of the data while maximizing the log density of the latent representation, as shown in Equation (6).

(6) $$\log p(x)=E_{z∼q\left(z|x\right)}\left[\log p\left(x|z\right)\right]-D_{KL}\left(q\left(z|x\right)∥p(z)\right)$$

Here, $D_{KL}\left(q\left(z|x\right)∥p(z)\right)$ refers to the Kullback–Leibler (KL) divergence, representing the difference between the variational distribution $q\left(z|x\right)$ and the original distribution p(z). When two distributions are exactly the same, the KL divergence is 0. A higher KL divergence value indicates a greater difference between the two distributions.

Training steps:

1. Utilize the encoder to map input data and random noise, obtaining hidden representations;
2. Employ the decoder to map the hidden representations back to the dimensions of the original data;
3. Calculate the logarithmic density of the hidden representations and maximize this objective.

We define three losses: (1) mean square error (MSE) loss between the actual image and the reconstructed image $\left(I_{t},{\hat{I}}_{t}\right)$; (2) MSE loss for gate pose reconstruction $\left(y_{t},{\hat{y}}_{t}\right)$; (3) KL divergence loss for each sample. 

### 2.4. Imitation Learning of the Control Policy

Imitation learning is a machine-learning method whose basic idea is to learn by observing and imitating the behavior of experts or known strategies. In imitation learning, the model attempts to learn the mapping relationship from input observations to output actions in order to achieve behavior performance similar to that of experts. Imitation learning mainly consists of three parts: firstly, the policy network; secondly, behavior demonstration (the continuous actions of experts); and thirdly, the environment simulator. To achieve the goal of imitation learning, this paper utilizes the behavior cloning method.

While it is often impossible to define a reward in many scenarios, it is possible to collect demonstration data from experts. For instance, in the underwater environment of the UE, it is not feasible to define rewards for robotic fish; however, records of numerous successful passages of gates by robotic fish can be collected. In the context of this paper, it typically involves expert robotic fish observing the current state of the environment at a given moment. The robotic fish then performs an action to pass through the gate in this state. After passing through the gate, the robotic fish enters the next state, where it performs another action. This sequence of states and actions is referred to as expert demonstration data.

**Figure 4:**
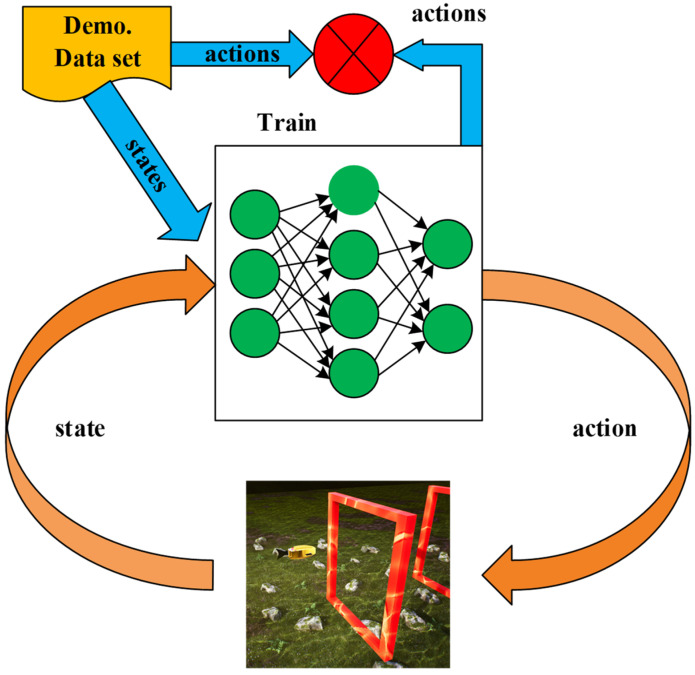
Imitation learning process.

Once the expert demonstration data are decomposed into state–action pairs, labeled data can be observed, meaning that for each state, the expert has performed a specific action. The intuitive idea of teaching the robotic fish to learn continuous actions is to use a form of supervised learning, where each state serves as a sample in the supervised learning framework and each action serves as a label. The state is treated as the input to the neural network, and the output of the neural network is treated as the action. Finally, by using the expected action, the robotic fish is taught to learn the corresponding relationship between states and actions. The process is illustrated in Figure 4.

In the above process, the training data are initially divided into training and validation sets. This partitioning process can be represented by blue arrows. Subsequently, the neural network is trained by minimizing the error on the training set until the error on the validation set converges [41]. Next, the trained neural network is applied to testing in the environment. The specific steps are as follows: Firstly, obtain the current state information from the environment. Then, utilize the trained neural network to determine the corresponding action. Finally, apply this action to the environment and observe its effects.

In reference to expert control policies and neural network control policies, following the approach of Rogerio et al. [42], this study designates $\pi^{*}(E)$ as the expert control policy. It seeks to find the optimal model parameters $\Theta^{*}$ and $\Phi^{*}$ in visual navigation. Under the observation state s of the expert, it minimizes the expected distance D between the control policy $\pi^{\Phi }\left(q_{RGB}^{\Theta }(I)\right)$ and the expert control policy $\pi^{*}(E)$, as shown in Equation (7).

(7) $$\Theta^{*},\Phi^{*}=\underset{\Theta ,\Phi }{\mathrm{argmin}}\;E_{s}\left[D\left(\pi^{*}(E),\pi^{\Phi }\left(q_{RGB}^{\Theta }(I)\right)\right]\right.$$

where the observation state s of the expert typically represents the environment state observed by the expert during task execution or the current state of the task. E defines the external environment, and $q_{RGB}^{\Theta }(I)$ encodes the input images.

To highlight the advantages of the cross-modal control strategy proposed in this paper, several contrasting strategies were introduced in the simulation experiments as follows:

The first one is the cross-modal control strategy $BC_{unc}$ introduced in this paper, which utilizes features called $z_{unc}$. These features are latent space representations extracted from multimodal data. This strategy is based on imitation learning, learning the mapping relationship from these features to the behavior of the robot fish from expert demonstration data.

The second one is the $BC_{img}$ strategy, which employs pure unsupervised image reconstruction VAE as features. This means that $BC_{img}$ directly uses images as inputs without first extracting other features from the images. $BC_{img}$ aims to learn the behavior strategy of the robot fish by learning the latent space representation of images. During training, $BC_{img}$ minimizes the difference between the original image and the reconstructed image to learn the effectiveness of image representation.

The third one is the $BC_{reg}$ strategy, which utilizes a purely supervised regressor, mapping from images to the gate pose as features. This means that $BC_{reg}$ does not directly learn the robot’s action strategy but attempts to predict the position of the gate. During training, $BC_{reg}$ uses the true position of the gate as a label and trains the model by minimizing the error between the predicted position and the actual position.

The fourth one is the $BC_{full}$ strategy, which employs full end-to-end mapping, directly mapping from images to velocities, without an explicit latent feature vector. This means that $BC_{full}$ directly learns the robot’s velocity control strategy from images without the need for intermediate feature representations. During training, $BC_{full}$ directly minimizes the error between the predicted velocity and the actual velocity.

## 3. Experimental Analysis and Validation

### 3.1. Experimental Environment

The experiment was carried out on the Windows 11 operating system, employing tools such as the UE (Unreal Engine), PyCharm, 3ds Max, and the Airsim plugin. The experimental hardware platform comprised a 13th Generation Intel(R) Core (TM) i7-13700 CPU running at 2.10 GHz, 32 GB of RAM, and an NVIDIA GeForce RTX 4060 GPU with 24 GB of dedicated memory.

**Figure 5:**
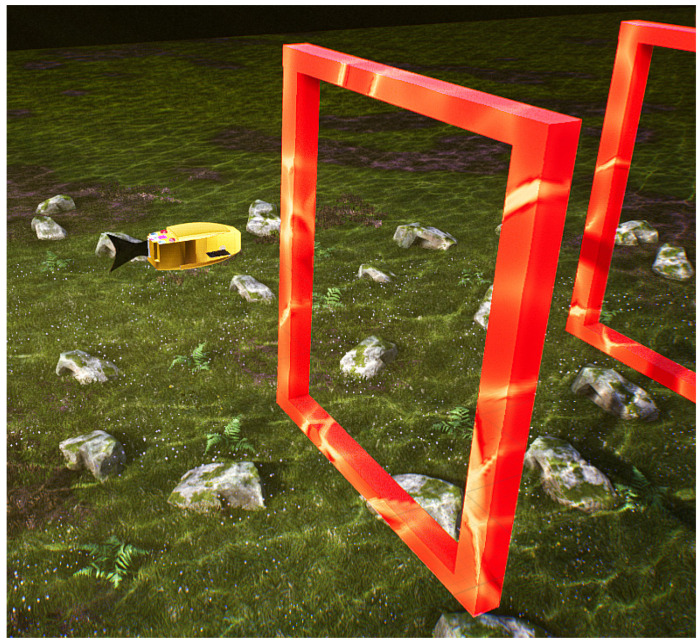
Model of the robot fish in the underwater environment of the UE.

The undersea environment necessary for the experiment was established within the UE simulation platform. The robotic fish model was developed using 3ds Max software, aiming to resemble the boxfish in look. Subsequently, the model was integrated into the aquatic environment of the UE simulation platform, as illustrated in Figure 5; the first gate is located 3 m directly in front of the robot fish.

### 3.2. Experimental Analysis

To assess different task modules, such as first-person vision detection, cross-modal VAE architecture, and simulation learning of control methods, on the UE simulation platform, we created several obstacle-crossing simulation scenarios in the underwater environment of the UE. The robotic fish successfully maneuvers through doorframe barriers using only visual navigation, without the need for communication.

#### 3.2.1. Different Track-Crossing Outcomes

(1) Straight-line track

**Figure 6:**
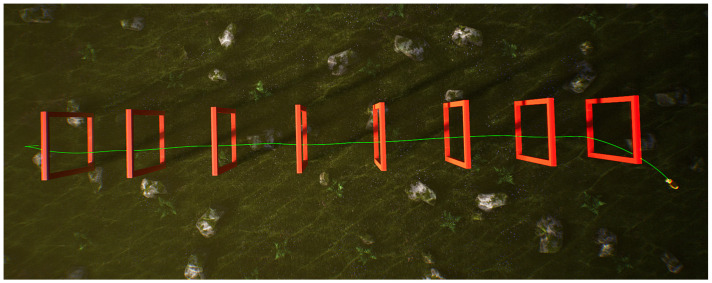
Single robot fish crossing the linear track.

In the underwater environment of the UE simulation platform, eight doorframes are arranged along a 50 m straight-line track, as shown in Figure 6. The initial position of the robotic fish is on the left side of the figure, and external images are obtained through the front camera of the robotic fish for navigation. The red doorframes in the figure represent the real poses for navigating through obstacles, and the green curve represents the swimming trajectory achieved by the fish through the $BC_{unc}$ control strategy. During the swimming process, the trajectory can be dynamically updated based on the detection information of the doorframe obstacles.

Through the analysis of the above figure, it can be observed that the robotic fish, starting from the left initial position, can smoothly navigate through these eight doorframe obstacles based on the first-person view, achieving a success rate of 100%. The green curve maintains a central position within the doorframes, and there is no collision with the doorframes. This validates that the proposed method in this paper can successfully navigate obstacles on a linear track. In order to ensure the adaptability of the proposed method in different scenarios, the doorframe obstacles are replaced with an S-shaped track.

(2) S-shaped curve track

**Figure 7:**
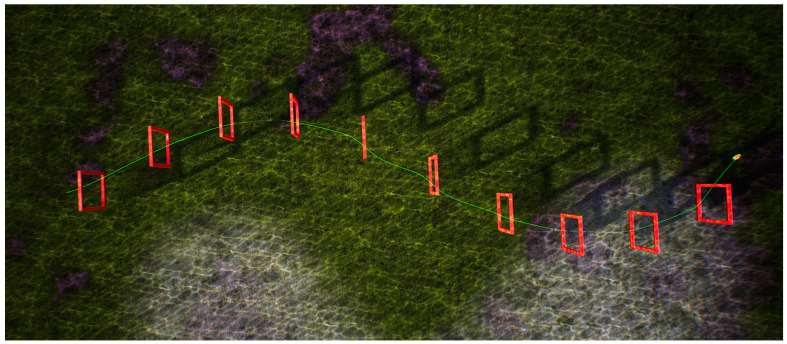
Single robot fish crossing the S-shaped curved track.

By placing 10 doorframes in the underwater environment of the UE, a 90 m long S-shaped curved track is established, as shown in Figure 7. The initial position of the robotic fish is on the left, and it navigates through the doorframe obstacles of the S-shaped track using the $BC_{unc}$ control strategy.

Upon analyzing the aforementioned figure, it is evident that the robotic fish is capable of navigating around the obstacles of the S-shaped track with ease, ultimately reaching the target without any deviation from the route. The probability of successfully navigating through the doorframes is 100%. While navigating, the robotic fish may encounter momentary friction with the inside surfaces of the doorframes. However, the visual navigation technique adapts the swimming direction in real-time to effectively maneuver around the difficulties posed by the doorframes in subsequent tries. This experiment confirms the practicality of the suggested visual navigation technique in navigating across curves with a S shape. In order to improve the flexibility of this approach in intricate surroundings, the doorframe obstructions are substituted by circular tracks.

(3) Circular Track

**Figure 8:**
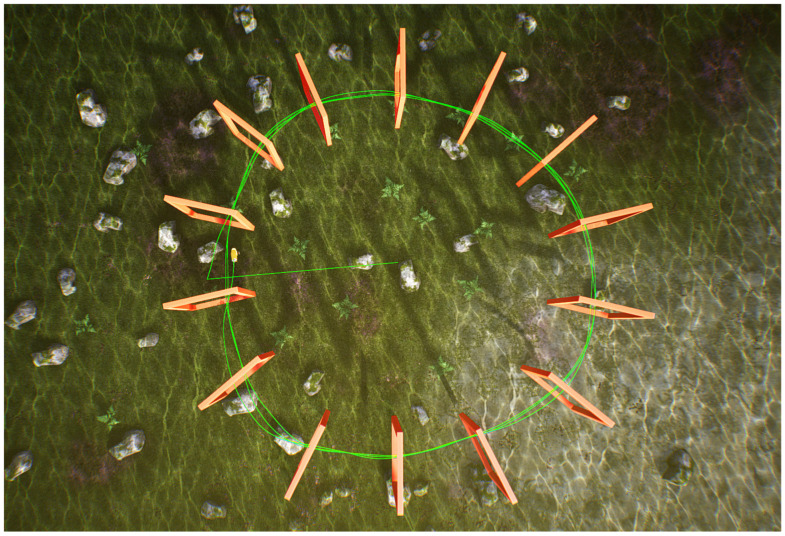
Single robot fish crossing the circular track.

In the underwater environment of UE, a circular track with a radius of 16 m was established, as shown in Figure 8. The circumference of the track is 100.48 m, and it includes 14 doorframes. The initial position of the robotic fish is at the center of the circular track. After starting the operation, it moves to the left circumference and then navigates through the circular doorframes using the $BC_{unc}$ control strategy.

After examining the figure, it can be inferred that the robotic fish is capable of navigating the circular doorframe track in the UE environment with ease. Friction and collision are absent when interacting with the doorframes, and the green trajectory line consistently aligns with the central area of the rectangular doorframes. The probability of successfully going through the doorframes is 100%. Furthermore, the robotic fish is instructed to complete three laps around the circular track, with each lap following a trajectory that maintains close proximity to the center of the rectangular doorframes. This ensures the stability and precision of the optical navigation technique. This confirms the effectiveness of the navigation method in successfully crossing doorframes on a circular path.

The robotic fish in the UE simulation platform has effectively demonstrated the capacity to navigate through doorframe barriers, including linear tracks, S-shaped curved tracks, and circular tracks. The robotic fish can pass through doorframes on various types of tracks by detecting the position and orientation of the doorframes and employing a flexible control technique. This suggests that inside a simulated environment, the visual navigation and control algorithms of the robotic fish demonstrate adaptability, efficiently overcoming problems presented by different track geometry and obstructions such as doorframes. This feature is crucial for allowing the robotic fish to execute intricate motion tasks in particular missions and situations.

#### 3.2.2. Effect of Multi-Robotic Fish Navigation

**Figure 9:**
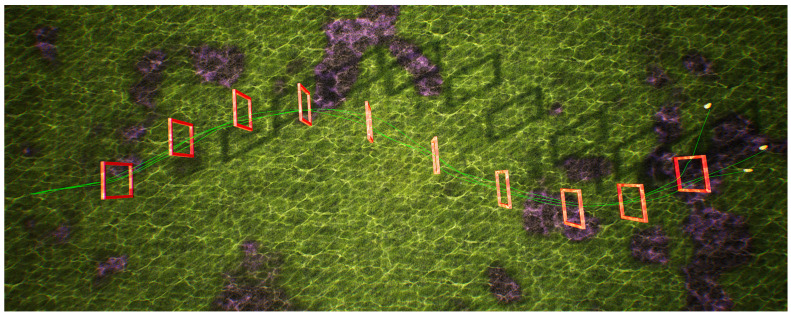
Multi-robotic fish navigating through the S-shaped gate track.

To validate the effectiveness of the visual navigation approach in multi-robotic fish navigating through gates, a simulated underwater environment was created in the UE simulation platform. A track with an S-shaped curve, comprising 10 gates and measuring 90 m in length, was established, as illustrated in Figure 9. Due to the increased difficulty and representativeness of navigating through S-shaped gates for multiple robotic fish compared to linear or circular gates, this configuration was selected for the study. The figure depicts three robotic fish, initially positioned on the left side of the S-shaped track. All three robotic fish employ the $BC_{unc}$ control strategy for navigating through gate obstacles, and the green curves represent the trajectories of the multi-robotic fish.

Upon examination of the depicted diagram, it is evident that the three robotic fish are capable of navigating the S-shaped gate track with ease. Furthermore, the trajectory of each fish, represented by the color green, remains inside the boundaries of the red gate frames. Given that the group of robotic fish were able to navigate the gate track 30 times using visual navigation control, without any deviations from the S-shaped trajectory, and achieved a 100% success rate, they fulfill the criteria for overcoming gate obstacles. This study evaluates the overall performance of several robotic fish navigating through gates by conducting experiments on the UE simulation platform. The assessment includes measures such as the number of successful traversals and the stability of the swimming trajectories. The purpose of this evaluation is to confirm the dependability of the visual navigation system in this particular task.

### 3.3. Experimental Comparison

For the experiment involving multiple robotic fish navigating obstacles, this study conducts comparative experiments based on four different types of control strategies. As mentioned earlier, behavior cloning strategy $BC_{unc}$ was trained on the latent space of CM-VAE, direct gate pose regressor $BC_{reg}$, conventional VAE image reconstruction feature $BC_{img}$, and finally, fully end-to-end trained $BC_{full}$.

**Figure 10:**
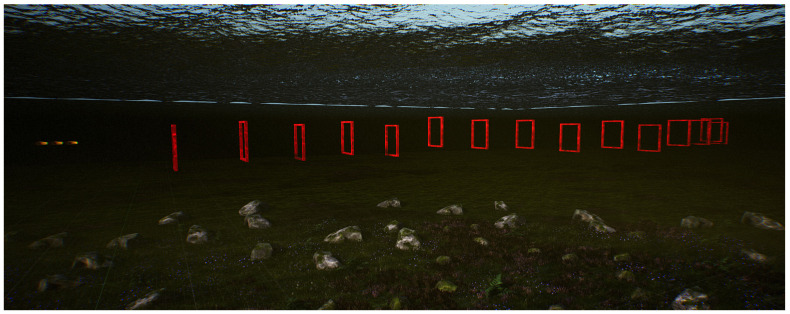
Noisy S-shaped track. The noisy track exhibits non-uniform height along the z-axis direction.

**Figure 11:**
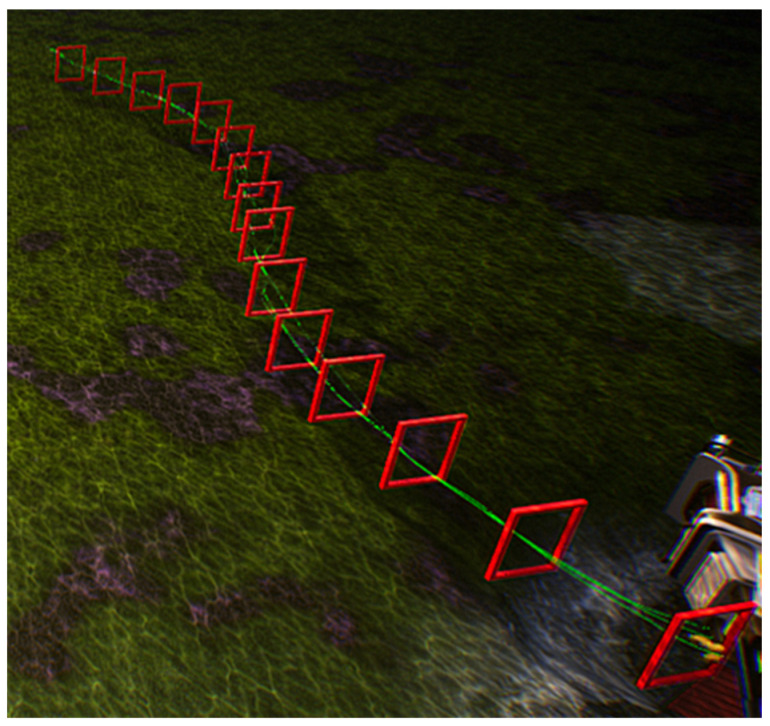
The schematic diagram of multiple robotic fish crossing the gate frame under the $BC_{unc}$ control strategy.

**Figure 12:**
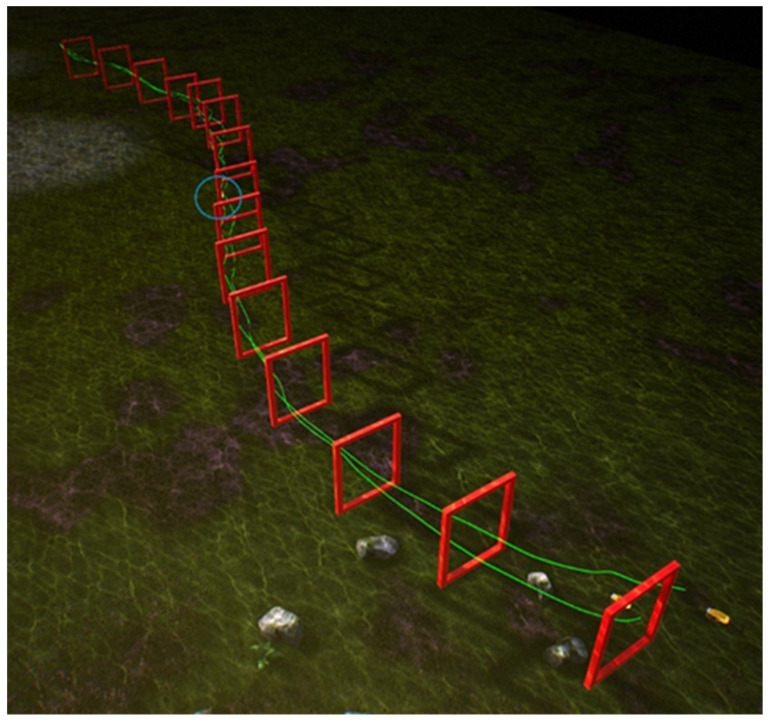
The schematic diagram of multiple robotic fish crossing the gate frame under the $BC_{reg}$ control strategy.

**Figure 13:**
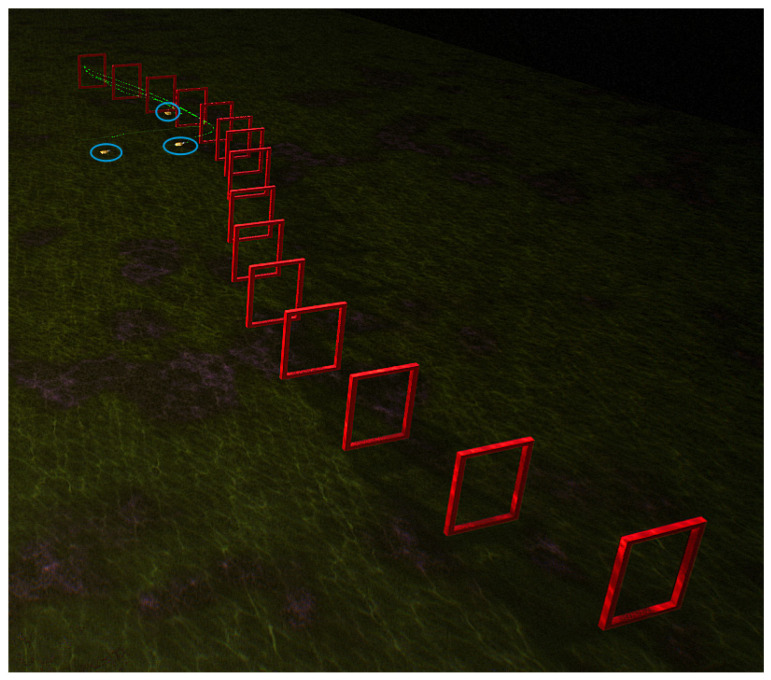
The schematic diagram of multiple robotic fish crossing the gate frame under the $BC_{full}$ control strategy.

**Figure 14:**
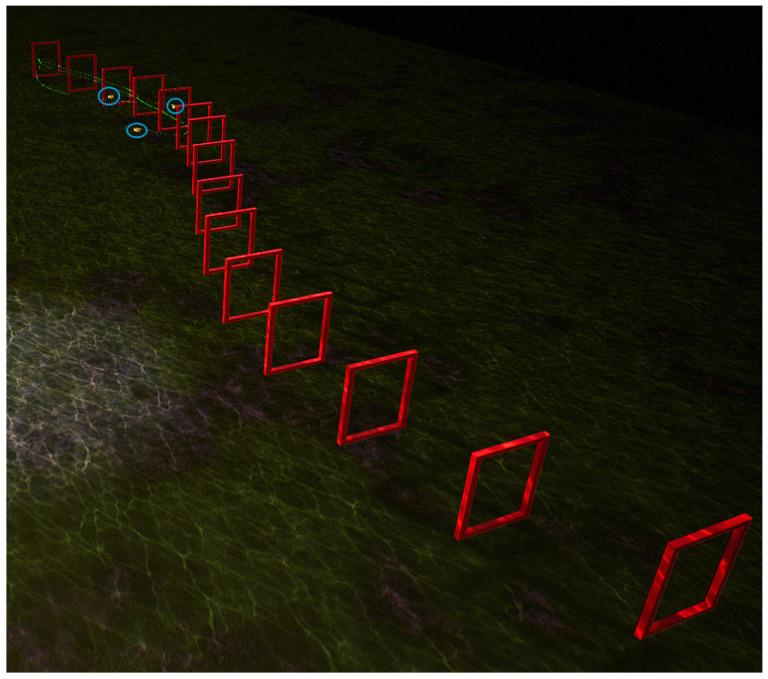
The schematic diagram of multiple robotic fish crossing the gate frame under the $BC_{img}$ control strategy.

To compare these four control strategies, this study established a simulation environment in the underwater setting of the UE platform, featuring a S-shaped curve track with 15 gates and a length of 140 m, as shown in Figure 10. In order to better highlight the distinguishability of these control strategies, random noise was added along the Z-axis direction of the obstacle gates, resulting in varying heights for each gate along the curved track, thereby increasing the difficulty of the robotic fish in navigating through the gates. Figure 11, Figure 12, Figure 13 and Figure 14 respectively depict schematics of crossing the gate frames under the four aforementioned control strategies.

From the above four diagrams, it can be observed that under the $BC_{unc}$ control strategy, multiple robotic fish can almost completely pass through all the gate frames. Under the $BC_{reg}$ control strategy, most of them can pass through the gate frames, but one robotic fish is stuck in the gate frame and unable to move within the blue circle in Figure 12. However, under the $BC_{full}$ and $BC_{img}$ control strategies, three robotic fish cannot successfully pass through all the gate frames, and some even deviate from the track.

To further the differentiation of these four control systems, several robotic fish were subjected to testing on an elongated S-shaped trajectory containing multiple gate frames. Every control technique was subjected to 10 experimental trials, with the criterion for success being a 100% traversal rate of all gate frames by the robotic fish. The control strategy tests yielded the following results, as shown in Table 1: three robotic fish required a total of 450 gate traversals. The success rate was obtained by dividing the number of gate traversals by the total count of 450.

Through analysis of the above table, it can be observed that the performance of multiple robotic fish under the control of the behavior cloning strategy $BC_{unc}$ is the most optimal. The number of gate traversals is 446, with only 4 gate frames remaining untraversed, resulting in a success rate of 98.89%. This strategy exhibits the highest performance among the analyzed control strategies. In comparison, the control performance of the $BC_{reg}$ strategy is relatively poor, with only 89.11% of gate frames successfully traversed; however, its performance surpasses that of the $BC_{full}$ and $BC_{img}$ strategies. Under the navigation control of $BC_{full}$ and $BC_{img}$, the robotic fish demonstrate the poorest performance in gate traversal, with success rates of only 18.00% and 16.22%, respectively, making it nearly impossible to complete a full experiment successfully.

Overall, the performance of the two architectures, $BC_{unc}$ and $BC_{reg}$, which encode gate positions, is significantly superior to the benchmark model. This may stem from the fact that gate frames represent pixel footprints in the overall image, making it challenging for conventional VAE architectures or end-to-end learning to effectively capture the gate frame orientations.

## 4. Discussion

Given the intricate nature of underwater settings, it is difficult to attain accurate control over several robotic fish via optical navigation. This paper presents a vision-based obstacle traversal strategy for numerous robotic fish as a solution to the problem at hand. The method integrates CM-VAE with imitation learning to facilitate the navigation of the robotic fish through gate frames. The control system framework for the robotic fish entails acquiring first-person view (FPV) using a camera component integrated into a user equipment UE. Afterwards, the FPV images are transformed into a lower-dimensional latent space using CM-VAE. The underlying characteristics in this domain are subsequently linked to the velocity instructions of the robotic fish using imitation learning, which is based on the control method.

To validate the effectiveness of the visual navigation method proposed in this study, experiments were conducted utilizing the $BC_{unc}$ control strategy for both single and multiple robotic fish traversing linear, S-shaped, and circular gate frame trajectories. The experimental results are presented in Figure 6, Figure 7, Figure 8 and Figure 9. Experimental analysis indicates that the robotic fish can smoothly and accurately traverse all gate frames on the trajectories.

Experiments were conducted on a noisy S-shaped gate frame trajectory for different control strategies, namely $BC_{unc}$, $BC_{reg}$, $BC_{img}$, and $BC_{full}$. The experimental results are presented in Table 1. Through comparative analysis of the experiments, it is evident that the $BC_{unc}$ control strategy yields the best performance, followed by $BC_{reg}$ with moderate effectiveness, while $BC_{img}$ and $BC_{full}$ exhibit the least favorable outcomes.

The obstacle traversal technique for multiple robotic fish, which utilizes CM-VAE and imitation learning, has made notable achievements but also encounters specific limitations. This offers essential guidance for future research endeavors. The constraints encompass the following:

(i) Restricted ecological adaptability: The technology’s capacity to function well in various situations may be limited when it is educated in certain settings. When placed in unfamiliar surroundings, a group of robotic fish may encounter difficulties in adjusting and efficiently navigating obstacles. Subsequent investigations may prioritize enhancing the model’s capacity to adjust to diverse environmental fluctuations;

(ii) Sample efficiency and data requirements: Imitation learning sometimes necessitates a substantial quantity of sample data to achieve optimal performance, a task that may be difficult to do in real-world scenarios. Future research endeavors may focus on improving the algorithm’s sample efficiency and decreasing reliance on extensive datasets;

(iii) Uncertainty management: Models may encounter difficulties in dynamic and uncertain contexts, particularly when navigating obstacles. Subsequent investigations may focus on strategies to manage and alleviate environmental unpredictability in order to improve the resilience of many robotic fish in intricate situations.

To overcome these constraints and propel the technology forward, potential avenues for future study may encompass the following:

(i) Reinforcement learning introduction: The integration of reinforcement learning processes allows for the ongoing improvement of strategies by interacting with the environment, resulting in enhanced adaptability and generalization performance;

(ii) Transfer learning methods: Employing transfer learning methods to enhance the application of knowledge gained in one setting to different settings hence enhances the algorithm’s adaptability;

(iii) Instantaneous decision-making and strategic planning: Highlighting the need to meet immediate time constraints and investigating more effective approaches to making decisions and creating plans is important in order to promptly address challenges in ever-changing settings.

By exploring these instructions, future study might enhance the obstacle traversal method for multiple robotic fish using cross-modal VAE and imitation learning, hence increasing its practicality and robustness.

## 5. Conclusions

This study presents a novel visual navigation method that integrates CM-VAE and imitation learning. The algorithm is implemented in the control system of a robotic fish to enable it to traverse gates in various surroundings. This research primarily aims to give the comprehensive foundation of the control system for robotic fish. The robotic fish acquires the first-person view (FPV) by utilizing the camera component in the UE. Afterwards, the images are transformed into a low-dimensional latent space using CM-VAE. The underlying characteristics in this domain are subsequently translated into the velocity instructions for the robotic fish using the control approach. The CM-VAE design utilizes distinct encoder–decoder pairs for each data modality, while restricting all inputs and outputs to a common latent space. The imitation learning for control approach employs behavioral cloning, which is a form of supervised learning. Ultimately, the utilization of the visual navigation method in robotic fish is showcased. By conducting trials with a solitary robotic fish navigating linear, S-shaped, and circular gate frame paths, as well as multiple robotic fish navigating an S-shaped gate frame path, it was determined that the control approach successfully traverses several types of gate frame paths with 100% stability and precision. In experiments on a noisy S-shaped gate frame trajectory with navigation using $BC_{unc}$, $BC_{reg}$, $BC_{img}$, and $BC_{full}$ control strategies, it is observed that $BC_{unc}$ outperforms the other strategies with an accuracy exceeding 98%.

Although significant progress has been made in the simulation experiments of multiple robotic fish crossing obstacles based on visual navigation, practical applications in real environments have not yet been realized. Robotic fish may encounter challenges in real environments, such as inadequate environmental adaptability, real-time requirements, and cost and resource demands. Future research may focus on enhancing the environmental adaptability and generalization capability of models, improving sensor technology, and optimizing cost and resource utilization efficiency.

## Figures and Tables

**Table 1:** Performance of various navigation strategies on simulated trajectory.

Control Strategy	${\mathrm{BC}}_{unc}$	${\mathrm{BC}}_{reg}$	${\mathrm{BC}}_{full}$	${\mathrm{BC}}_{img}$
Number of Gate Traversals	446	401	81	73
Success Rate	98.89%	89.11%	18.00%	16.22%

## Data Availability

Some or all of the data, models, or code that support the findings of this study are available from the corresponding author upon reasonable request.

## References

[1] Marroquín A., Garcia G., Fabregas E., Aranda-Escolástico E., Farias G. (2023). Mobile Robot Navigation Based on Embedded Computer Vision. Mathematics.

[2] Dilek E., Dener M. (2023). Computer vision applications in intelligent transportation systems: A survey. Sensors.

[3] Dobler G., Vani J., Dam T.T.L. (2021). Patterns of urban foot traffic dynamics. Comput. Environ. Urban Syst..

[4] Wang X.T., Fan X.N., Shi P.F., Ni J.J., Zhou Z.K. (2023). An Overview of Key SLAM Technologies for Underwater Scenes. Remote Sens..

[5] Chang Z., Wang M., Wei Z., Yu J.Z. A Bionic Robotic Fish Detection Method by Using YOLOv3 Algorithm. Proceedings of the 2020 Chinese Automation Congress (CAC).

[6] López-Barajas S., González J., Sandoval P.J., Gómez-Espinosa A., Solis A., Marín R., Sanz P.J. Automatic Visual Inspection of a Net for Fish Farms by Means of Robotic Intelligence. Proceedings of the 2023 IEEE OCEANS 2023-Limerick.

[7] Wang F., Zhang C.F., Zhang W., Fang C.Y., Xia Y.W., Liu Y., Dong H. (2022). Object-Based Reliable Visual Navigation for Mobile Robot. Sensors.

[8] Rao Z.H., Wu Y., Yang Z.F., Zhang W., Lu S.J., Lu W.Z., Zha Z.J. (2021). Visual navigation with multiple goals based on deep reinforcement learning. IEEE Trans. Neural Netw. Learn. Syst..

[9] Karnan H., Warnell G., Xiao X., Stone P. Voila: Visual-observation-only imitation learning for autonomous navigation. Proceedings of the 2022 IEEE International Conference on Robotics and Automation (ICRA).

[10] Qin J.Y., Li M., Li D.R., Zhong J.G., Yang K. (2022). A survey on visual navigation and positioning for autonomous UUVs. Remote Sens..

[11] Cong P.C., Liu J.J., Li J.X., Xiao Y.X., Chen X.L., Feng X.J., Zhang X. (2023). YDD-SLAM: Indoor Dynamic Visual SLAM Fusing YOLOv5 with Depth Information. Sensors.

[12] Wang Y., Zhang Y., Hu L.H., Wang W., Ge G.Y., Tan S.Y. (2023). A Semantic Topology Graph to Detect Re-Localization and Loop Closure of the Visual Simultaneous Localization and Map** System in a Dynamic Environment. Sensors.

[13] Dall’Osto D., Fischer T., Milford M. Fast and robust bio-inspired teach and repeat navigation. Proceedings of the 2021 IEEE International Conference on Intelligent Robots and Systems (IROS).

[14] Rozsypalek Z., Broughton G., Linder P., Roucek T., Blaha J., Mentzl L., Kusumam K., Krajník T. (2022). Contrastive learning for image registration in visual teach and repeat navigation. Sensors.

[15] Camara L.G., Pivonka T., Jílek M., Gäbert C., Kosnar K., Preucil L. Accurate and robust teach and repeat navigation by visual place recognition: A CNN approach. Proceedings of the 2020 12th IEEE/RSJ International Conference on Intelligent Robots and Systems (IROS).

[16] Roucek T., Amjadi A.S., Rozsypálek Z., Broughton G., Blaha J., Kusumam K., Krajník T. (2022). Self-Supervised Robust Feature Matching Pipeline for Teach and Repeat Navigation. Sensors.

[17] Zhang W.Z., He L., Wang H.W., Yuan L., Xiao W.D. (2023). Multiple Self-Supervised Auxiliary Tasks for Target-Driven Visual Navigation Using Deep Reinforcement Learning. Entropy.

[18] Djenouri Y., Hatleskog J., Hjelmervik J., Bjorne E., Utstumo T., Mobarhan M. (2022). Deep learning based decomposition for visual navigation in industrial platforms. Appl. Intell..

[19] Vijetha U., Geetha V. (2023). Optimizing Reinforcement Learning-Based Visual Navigation for Resource-Constrained Devices. IEEE Access..

[20] Liu S.P., Tian G.H., Shao X.Y., Liu S. (2022). Behavior Cloning-Based Robot Active Object Detection with Automatically Generated Data and Revision Method. IEEE Trans. Robot..

[21] Ashlesha A., Lin C.T. (2021). Time and Action Co-Training in Reinforcement Learning Agents. Front. Control. Eng..

[22] Guo K., Pan Y.P., Zheng D.D., Yu H.Y. (2020). Composite learning control of robotic systems: A least squares modulated approach. Automatica.

[23] Wu T., Zhou P., Liu K., Yuan Y., Wang X., Huang H., Wu D.O. (2020). Multi-agent deep reinforcement learning for urban traffic light control in vehicular networks. IEEE Trans. Veh. Technol..

[24] Carrillo Mendoza R. (2021). Deep Learning-Based Localisation for Autonomous Vehicles. Ph.D. Thesis.

[25] Bozek P., Karavaev Y.L., Ardentov A.A., Yefremov K.S. (2020). Neural network control of a wheeled mobile robot based on optimal trajectories. Int. J. Adv. Robot. Syst..

[26] Šegota S.B., Anđelić N., Mrzljak V., Lorencin I., Kuric I., Car Z. (2021). Utilization of multilayer perceptron for determining the inverse kinematics of an industrial robotic manipulator. Int. J. Adv. Robot. Syst..

[27] Garrote L., Torres M., Barros T., Perdiz J., Premebida C., Nunes U.J. Mobile robot localization with reinforcement learning map update decision aided by an absolute indoor positioning system. Proceedings of the 2019 IEEE/RSJ International Conference on Intelligent Robots and Systems (IROS).

[28] Gao X.L., Luo H.Y., Ning B.K., Zhao F., Bao L.F., Gong Y.L., Xiao Y.M., Jiang J.G. (2020). RL-AKF: An Adaptive Kalman Filter Navigation Algorithm Based on Reinforcement Learning for Ground Vehicles. Remote Sens..

[29] Spurr A., Song J., Park S., Hilliges O. Cross-modal deep variational hand pose estimation. Proceedings of the 2018 IEEE Conference on Computer Vision and Pattern Recognition.

[30] Wu Q.Y., Gong X.X., Xu K., Manocha D., Dong J.X., Wang J. (2020). Towards target-driven visual navigation in indoor scenes via generative imitation learning. IEEE Robot. Autom. Lett..

[31] Yan C.Z., Qin J.H., Liu Q.C., Ma Q.C., Kang Y. (2022). Mapless navigation with safety-enhanced imitation learning. IEEE Trans. Ind. Electron..

[32] Deng Y., Xu K., Hu Y., Cui Y., Xiang G., Pan Z. Learning Effectively from Intervention for Visual-based Autonomous Driving. Proceedings of the 2022 IEEE 25th International Conference on Intelligent Transportation Systems (ITSC).

[33] Baran B., Krzyzowski M., Rádai Z., Francikowski J., Hohol M. (2023). Geometry-based navigation in the dark: Layout symmetry facilitates spatial learning in the house cricket, Acheta domesticus, in the absence of visual cues. Anim. Cogn..

[34] Arora S., Doshi P. (2021). A survey of inverse reinforcement learning: Challenges, methods and progress. Artif. Intell..

[35] Xu Z., Wang S., Li K. Goal Conditioned Generative Adversarial Imitation Learning Based on Dueling-DQN. Proceedings of the International Conference on Autonomous Unmanned Systems.

[36] Zhang X., He B., Li G.L., Mu X.K., Zhou Y., Mang T.J. (2020). NavNet: AUV navigation through deep sequential learning. IEEE Access.

[37] Ruscio F., Tani S., Bresciani M., Caiti A., Costanzi R. (2022). Visual-based navigation strategy for autonomous underwater vehicles in monitoring scenarios. FAC-PapersOnLine.

[38] Song J.B., Li W.Q., Zhu X.W. (2023). Acoustic-VINS: Tightly Coupled Acoustic-Visual-Inertial Navigation System for Autonomous Underwater Vehicles. IEEE Robot. Autom. Lett..

[39] Yan S.Z., Wang J., Wu Z.X., Tan M., Yu J.Z. (2023). Autonomous vision-based navigation and stability augmentation control of a biomimetic robotic hammerhead shark. IEEE Trans. Autom. Sci. Eng..

[40] Wei Y., Levesque J.P., Hansen C.J., Mauel M.E. (2021). A dimensionality reduction algorithm for mapping tokamak operational regimes using a variational autoencoder (VAE) neural network. Nuclear Fusion..

[41] Najar A., Bonnet E., Bahrami B., Palminteri S. (2020). The actions of others act as a pseudo-reward to drive imitation in the context of social reinforcement learning. PLoS Biol..

[42] Bonatti R., Madaan R., Vineet V., Scherer S., Kapoor A. Learning visuomotor policies for aerial navigation using cross-modal representations. Proceedings of the IEEE Conference on International Conference on Intelligent Robots and Systems (IROS).

